# Preprosthetic Surgery—Narrative Review and Current Debate

**DOI:** 10.3390/jcm12237262

**Published:** 2023-11-23

**Authors:** Hendrik Terheyden, Gerry M. Raghoebar, Mats Sjöström, Thomas Starch-Jensen, John Cawood

**Affiliations:** 1 Department of Oral & Maxillofacial Surgery, Helios Hospitals, 34121 Kassel, Germany; 2 Deptartment of Oral & Maxillofacial Surgery, University of Groningen, University Medical Center Groningen, 9713 GZ Groningen, The Netherlands; g.m.raghoebar@umcg.nl; 3 Oral and Maxillofacial Surgeon, Umeå University, 901 87 Umeå, Sweden; mats.sjostrom@umu.se; 4 Department of Oral and Maxillofacial Surgery, Aalborg University Hospital, 9000 Aalborg, Denmark; thomas.jensen@rn.dk; 5 Emeritus Consultant Oral and Maxillofacial Surgeon, Mersey Regional Health Authority, UK; johnicawood@gmail.com

**Keywords:** alveolar bone atrophy, alveolar bone loss, bone grafting, dental implants, edentulous jaw, preprosthetic oral surgery procedures

## Abstract

This review describes the role of modern preprosthetic surgery. The atrophic edentulous jaw can cause severe functional impairment for patients, leading to inadequate denture retention, reduced quality of life, and significant health problems. The aim of preprosthetic surgery is to restore function and form due to tooth loss arising from congenital deformity, trauma, or ablative surgery. Alveolar bone loss is due to disuse atrophy following tooth loss. The advent of dental implants and their ability to preserve bone heralded the modern version of preprosthetic surgery. Their ability to mimic natural teeth has overcome the age-old problem of edentulism and consequent jaw atrophy. Controversies with preprosthetic surgery are discussed: soft tissue versus hard tissue augmentation in the aesthetic zone, bone regeneration versus prosthetic tissue replacement in the anterior maxilla, sinus floor augmentation versus short implants in the posterior maxilla—interpositional bone grafting versus onlay grafts for vertical bone augmentation. Best results for rehabilitation are achieved by the team approach of surgeons, maxillofacial prosthodontists/general dentists, and importantly, informing patients about the available preprosthetic surgical options.

## 1. Introduction

This review article discusses the role of contemporary preprosthetic surgery in managing the consequences of tooth loss, which culminates in the loss of oral function and facial form, and as a result, quality of life and self-esteem are affected. The lack of denture stability causes difficulty when eating in public. Furthermore, these problems are compounded by facial changes, such as a decrease in lower facial height, drooping chin, inverted lips, peri-oral wrinkling, and drooling. The aim of preprosthetic surgery is to improve denture stability and mastication whilst reducing jaw resorption, mucosal irritation fibromata, and possible dysplasia.

The term preprosthetic surgery was coined in the 1960s by Professor Hugo Obwegeser. Subsequently, in the 1980s, the International Research Group for Preprosthetic Surgery (IRGPS, now renamed the International Academy for Oral and Facial Rehabilitation “www.iaofr.org (accessed on 21 November 2023)”) was founded by John Cawood, Franz Härle, Eric Hjorting-Hansen, Hans de Koomen, Soren Hillerup, Hermann Sailer, Paul Stoelinga, Bill Terry, et al. [1], laid the foundation for contemporary preprosthetic surgery involving relative heightening of the edentulous ridge by soft tissue surgery (vestibuloplasty to increase the area of fixed mucosa) and absolute heightening of the edentulous ridge by bone grafting [2,3] including osteotomies (sandwich and visor in the mandible and Le Fort 1 in the maxilla) to increase denture retention. Unfortunately, until the advent of dental implants, these methods only provided temporary success as further resorption occurred due to denture-induced pressure [4].

Alveolar bone develops with tooth formation during facial growth. Consequently, following tooth loss, the alveolar bone resorbs due to disuse atrophy. Thus far, no pharmacological or surgical means, including socket preservation [5], has been found to prevent this physiological process other than the occlusal forces delivered by natural teeth. However, the realization that endosseous implants replacing natural teeth also prevent resorption by their ability to transmit functional loading internally forms the basis for predictable contemporary preprosthetic surgery. Grafted bone remains stable when subjected to functional loading by endosteal implants [6].

Jawbone regeneration in combination with dental implants is more than just enabling a dental prosthesis; it offers functional and aesthetic maxillofacial rehabilitation and has superseded tissue replacement by a plastic prosthesis, at least in younger patients. Nowadays, survival of natural teeth is the norm, rendering edentulism rare unless congenital absence, disease, trauma, or ablation intervenes, in which case oral rehabilitation is indicated. Not all patients can benefit from state-of-the-art preprosthetic surgery for health or economic reasons or due to the burden of surgery. Even so, other less invasive treatments involving more bulky prosthetic replacement aided by dental implants can suffice. The best results for rehabilitation are achieved by the team approach of surgeons, maxillofacial prosthodontists/general dentists, and, importantly, informing patients about the available preprosthetic surgical options.

Current debate in preprosthetic surgery tends to focus on attempting to reduce the amount of surgery in order to widen the range of providers and reduce costs for the patient. Such debate includes: the repair of small defects, especially in the aesthetic zone with bone grafts versus soft tissue alone? The repair of larger defects in the aesthetic zone with bone grafts instead of artificial prosthetic means? Can short implants in the posterior maxilla avoid the need for sinus floor augmentation? In implant dentistry, are onlay or interpositional grafts superior for vertical augmentation?

## 2. Soft versus Hard Tissue Augmentation in the Anterior Region

In this section, the indications for hard versus soft tissue augmentation needed for a favorable outcome of implant-based prosthodontics in the anterior region are described. The focus is on procedures to reconstruct a deficient alveolar ridge with bone and compromised soft tissue with soft tissue grafts to obtain the best functional and aesthetic outcome based on the literature.

Implant treatment in the aesthetic zone is a highly reliable option for the rehabilitation of failing or missing teeth [7]. With the demand for the most satisfying aesthetic outcome, the focus has shifted from just implant survival to enhancement and preservation of the hard and soft peri-implant tissues.

The alveolar ridge is subject to continual change, both horizontally and vertically, following tooth extraction [8], especially in the pre-maxilla. Therefore, placing dental implants in the aesthetic zone can be challenging as there is often a pre-existing deficiency of the alveolar ridge, which tends to compromise an aesthetic result. Augmenting the bone and soft tissue before or during implant placement can improve peri-implant conditions, but the timing and nature of any intervention to restore bone and soft tissue volume remains a matter of debate.

Bone augmentation is clearly indicated when an implant is incompletely covered by bone. It may be necessary to apply bone grafts and/or guided bone regeneration (GBR) to create enough bone volume. Guided bone regeneration is a well-documented technique and is effective in reducing bone defects that remain after implant placement. The aim of GBR is the establishment of a facial bone wall of sufficient thickness and height to support the soft tissues. Long-term GBR studies, in combination with implant placement, have demonstrated that thick and stable buccal bone walls can be achieved after lateral bone augmentation [9,10]. However, GBR can be invasive, involving at least one vertical releasing incision as well as a release of the periosteum and muscle insertion, increasing morbidity and mucosal scarring along the releasing incision(s) [11]. Fortunately, besides GBR, a plethora of other options are available to obtain a good bony foundation for an implant. A randomized clinical study was performed to compare GBR with a subepithelial connective tissue graft to increase the buccal soft tissue profile of single implant sites with a minor horizontal alveolar defect. At a three-year follow-up, no difference in the linear increase in buccal soft tissue profile was observed after either technique except for mucosal scarring, which was lower after subepithelial connective tissue grafting [12].

Even if there is no bone defect and no need for a bone graft, there may be a need for soft tissue grafting to improve the thickness and contour of the facial mucosa. Grafting of the peri-implant soft tissues can be performed before, simultaneously with implant placement, during osseointegration of the implant, or after prosthetic reconstruction [13]. Inadequate soft tissue dimensions can result in aesthetic and functional complications, oral hygiene maintenance, phonetic impediments, and susceptibility to mucosal recession.

**Figure 1:**
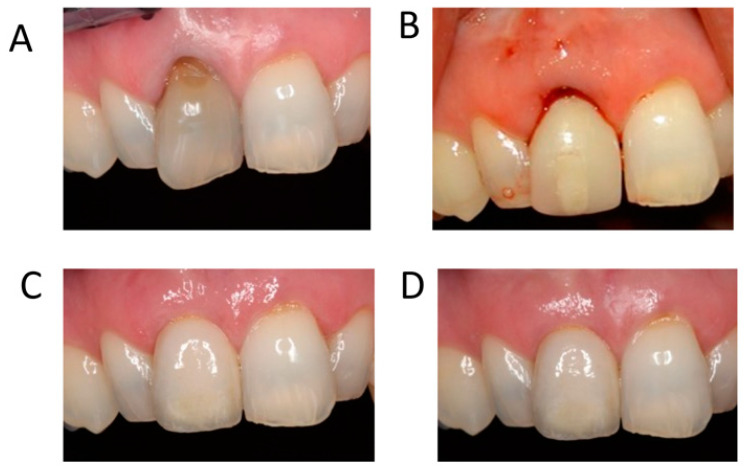
Immediate implant placement with immediate provisionalization and soft tissue grafting. (**A**) Clinical situation preoperative with failing 11 and thin mucosa. (**B**) Clinical situation after implant placement in the grafted socket without flap elevation, soft tissue grafting on the labial side, and placement of the screw-retained provisional crown. The tooth was removed in the same session. (**C**) Clinical situation after 1 year. (**D**) Clinical situation after 5 years. Nice contour and healthy aspect of the mucosa comparable to the neighboring teeth.

Soft tissue augmentation has also been proposed to re-establish buccal convexity. This approach can be considered when there is sufficient bone for complete embedment of the implant shoulder as well as when minor buccal bone defects causing soft tissue concavities are present [13,14,15]. The outcome of soft tissue augmentation is clinically relevant because thicker tissues enhance peri-implant health [14] and result in higher patient satisfaction and superior aesthetics [16]. Soft tissue augmentation in the aesthetic zone has been shown to result in less recession and a thicker mid-buccal mucosa following immediate implant placement and in less recession in the mid-buccal mucosa following delayed implant placement compared to no graft (Figure 1A–D) [13]. Therefore, soft tissue grafting has become a frequent adjunctive procedure for dental implant sites to enhance the quality and/or quantity of the mucosa [17]. Furthermore, mucosal thickness augmentation can mask the tissue discoloration caused by metallic abutments when the peri-implant mucosa is thin (<2 mm), and soft tissue augmentation appears to improve the stability of the mucosal margin [18]. Finally, soft tissue augmentation seems to enhance soft tissue stability in the vertical dimension, regardless of the timing of the implant placement [13].

The materials used for soft tissue grafting can be classified into three categories: (1) subepithelial connective tissue grafts harvested from the patients’ palates or tuberosities, (2) allografts, and (3) xenografts. Soft tissue substitutes (a volume-stable collagen matrix) resulted in similar stable peri-implant tissues, favorable aesthetics, and clinically negligible contour changes at 5 years post-loading than after using a subepithelial connective tissue graft [18]. However, in another study, the sites treated with a volume-stable collagen matrix demonstrated more shrinkage and significantly more marginal bone loss than those treated with a subepithelial connective tissue graft after 1 year [19]. Patient-reported outcome measures following soft tissue augmentation with soft tissue substitutes indicate a reduction in pain, less need for painkillers, and shorter surgery time while achieving similar patient satisfaction levels compared with autogenous grafts without impairing the clinical outcomes [17]. A longer follow-up is needed to determine the effectiveness and safety of the tissue substitutes; in particular, monitoring the marginal bone loss is crucial.

In summary, bone augmentation is needed to reconstruct a deficient ridge to a form that will ensure that an aesthetic implant-supported restoration with a good aesthetic outcome can be fabricated. Soft tissue augmentation is needed to improve the functional and aesthetic outcomes if there is any compromise in soft tissue thickness or quantity. A major limitation of most studies is the lack of long-term results.

## 3. Bone Reconstruction of the Anterior Maxilla

This section focuses on dentoalveolar reconstruction in the anterior maxilla. Tissue defects in the aesthetic area can be compensated by prosthetic means like artificial gingiva. However, knowledge of surgical procedures, bone remodeling, and osseointegration helps us to find a regenerative solution. When analyzing long-term prognosis and aesthetical results, the use of autogenous bone grafting and alveolar ridge reconstruction is the first choice, provided that the patient is fit for surgery. The aim is that the alveolar bone is adequate in height and width as well as providing support for a natural gingiva covering.

Replacement of the dentition in the anterior maxilla is demanding aesthetically, biologically, and biomechanically due to limited alveolar bone volume [20,21]. Trauma of the anterior maxilla is the major cause of the loss of a central incisor [22], whereas congenital absence is responsible for the majority of missing lateral incisors [23]. Disease, trauma, and tumor ablation are responsible for larger defects.

The buccal bone thickness in the anterior maxilla seldom exceeds one millimeter [24], and coupled with a further loss of about 50% during the healing process [25], compensation for ridge reduction is required. Avila-Ortiz et al. [26] evaluated techniques for alveolar ridge preservation and reported nine different ridge preservation treatment methods to reduce ridge resorption after tooth extraction. Although alveolar ridge preservation is positive, no superior method was found.

If the preoperative clinical and radiographic examination indicates insufficient alveolar bone volume, usually, two treatment options can be offered: prosthetic replacement with artificial gingiva (pink porcelain) or with a removable denture. In contrast, pre-implant bone reconstruction provides support for endosteal implants and support for the upper lip and a natural gingival appearance [27,28,29,30,31,32]. Each of these approaches reports good predictable results, but outcomes of bone reconstruction are technique-sensitive. The autogenous bone graft placed prior to implant surgery is widely reported [28,29,33]. One important factor for a predictable result after bone reconstruction is the degree of postoperative graft volume change that takes place during initial healing and remodeling. Sjöström et al. [34] found a post-grafting volume reduction of approximately one-third of the original bone volume in edentulous maxillae reconstructed with free iliac crest grafts. Approximately the same amount of graft reduction is reported with autogenous bone grafts from the mandibular ramus [35,36]. In a systematic review based on lateral ridge augmentation, Elnayef et al. [37] concluded that the estimated overall net bone gain at final re-evaluation was 2.86 ± 0.23 mm. The authors concluded that regardless of the material used for regeneration, different degrees of graft resorption should be expected. Block grafts seem to maintain the highest volume, and the authors recommended overcorrection of the horizontal defects to compensate for the resorption of the grafting materials. Troeltzsch et al. [38] concluded in a systematic review that horizontal and vertical gain by 3.7 mm on average can be achieved using particulate materials. In situations with extensive trauma resulting in the loss of several teeth and alveolar bone or resections due to tumors, more extensive reconstruction requires the use of extraoral bone block grafts. Starch-Jensen et al. [39] analyzed the implant survival after horizontal ridge augmentation with allogeneic bone block compared with autogenous bone block. No differences were found, but allogenic bone increased the risk for complications.

In severe vertical discrepancies, distraction osteogenesis can be used for reconstruction in the anterior maxilla, but the result compared to onlay bone grafting in terms of bone gain and bone resorption is questionable [40]. Zhao et al. [41] performed a systematic review of vertical distraction osteogenesis and concluded that vertical alveolar defects could be repaired successfully with distraction osteogenesis, and the implant placed in the distraction sites showed a high cumulative survival rate. However, the high complication rate necessitates caution. In situations with malignancies in the anterior maxilla, irradiation may be required. Irradiation impairs blood supply to the alveolar bone [42], which further compounds treatment. If implant treatment is decided, a minimally invasive surgical technique is mandatory [43]. In the event flapless surgery can reduce the risk of tissue complications. Flapless surgery for implant placement is reported to reduce peri-implant bone loss, compared to implant placement with flap preparation [44,45], but this finding is questioned by Lemos et al. [46], who found no significant differences for implant survival, changes in marginal bone level, or complications between the two techniques.

Marginal soft tissue is also important in the reconstruction of the anterior maxilla. Bienz et al. [47] concluded that increased soft tissue thickness at implant sites was associated with more favorable aesthetic outcomes. Hosseini et al. [48] compared connective tissue grafts harvested from the palate with a control group after both groups had received an implant in the anterior maxilla. The authors concluded that augmentation using a connective tissue graft may result in better mucosal color match and more facial dimensional gain compared to implant sites without soft tissue grafting. In situations with localized severe maxillary anterior ridge deformities, e.g., tumor resection or severe localized trauma, achieving a satisfactory aesthetic result is challenging. Mathews [49] evaluated three different soft tissue augmentation techniques and found that all three techniques demonstrated long-term stability following the six-month post-treatment result. These studies indicate that soft tissue grafts can improve the aesthetic result. Titanium placed in the aesthetic zone can achieve a successful result functionally even if the clinician is more critical of aesthetic outcomes than the patients [50].

**Figure 2:**
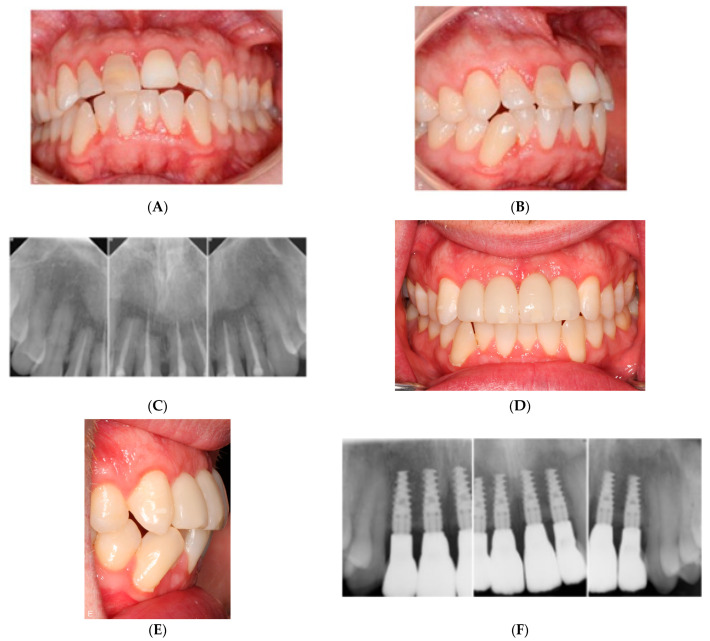
Trauma case. Teeth 12–22 avulsed due to bicycle trauma. Regio 12–22 reconstructed with zirconia crowns placed on four titanium implants (Nobel Active NP, 11.5 mm) using a virtual planning technique. (**A**) Anterior maxilla frontal view, preoperative status. (**B**) Anterior maxilla lateral view, preoperative status. (**C**) Intraoral radiographs region 12–22, preoperative status. (**D**) Anterior maxilla frontal view, postoperative status. (**E**) Anterior maxilla lateral view, postoperative status. (**F**) Intraoral radiographs region 12–22, postoperative status.

Implant placement can also be challenging in the anterior maxilla because of limited interdental space and bone volume, especially in single tooth situations. D’haese et al. [51] and Laederach et al. [52] discussed the importance of accuracy in critical anatomical situations and concluded that knowledge of implant deviation from the planned implant placement is important when using guided surgery. Åkesson et al. [53] analyzed virtually planned and flapless placed implants and concluded that the actual position of the implant placement compared to the virtually planned position often differed in the sagittal or vertical dimension, which should be considered when the available alveolar bone for the implant is severely limited, e.g., in the anterior maxilla. Figure 2A–F describes a case where the aesthetic zone is repaired with titanium implants (Nobel Active NP, 11.5 mm) and Zirconia crowns using the virtual planning technique.

## 4. Sinus Floor Augmentation and Standard-Length Implants versus Short Implants

Prosthetic rehabilitation of the moderately atrophic posterior maxilla with an implant-supported restoration is traditionally solved by grafting of the sinus floor for accommodation of standard-length dental implants. This section presents the current knowledge, which indicates that situations of maxillary sinus floor augmentation can be avoided by the use of short implants.

**Figure 3:**
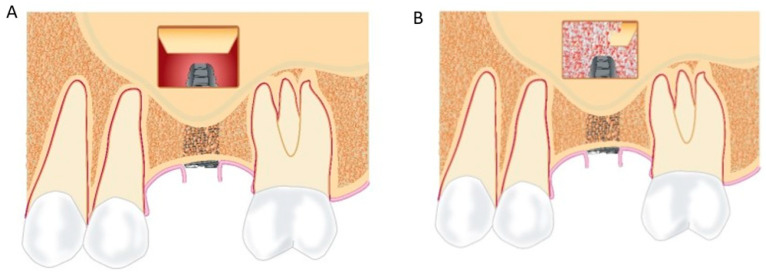
(**A**) The lateral bony window and the Schneiderian membrane are elevated from the original maxillary sinus floor, and a standard-length implant is inserted. The inserted implant is partially visible in the created compartment. (**B**) A grafting material is packed around the implant in the created compartment.

Maxillary sinus floor augmentation applying the lateral window technique and placement of a grafting material underneath the elevated Schneiderian membrane is frequently used to increase the alveolar ridge height of the atrophic posterior maxilla (Figure 3A,B). High survival rates of superstructures and implants, bone regeneration, limited peri-implant marginal bone loss, and low frequency of complications have been reported in systematic reviews and meta-analyses [54,55,56,57]. Autogenous bone graft generates the highest amount of new bone formation compared with other grafting materials in conjunction with sinus floor augmentation [58,59]. However, the use of autologous bone grafts is associated with supplementary surgery, risk of donor site morbidity, and an unpredictable resorption of the augmented volume [60,61,62,63]. Autologous bone is, therefore, often combined or replaced by bone substitutes to simplify the surgical procedure. Bone substitutes solely provide osteoconductive properties and contain a theoretical risk of disease transmission. Thus, from a clinical and patient perspective, it would be a significant improvement if implant placement in the atrophic posterior maxilla could be performed without maxillary sinus floor augmentation and a graft.

**Figure 4:**
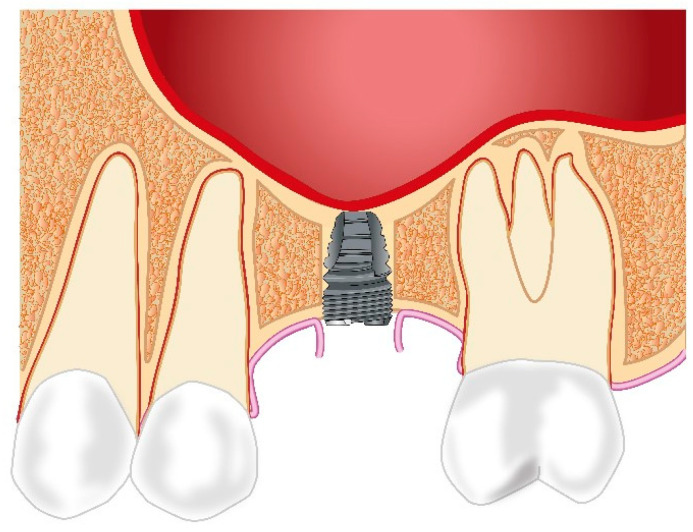
A short implant is inserted in the posterior maxilla avoiding maxillary sinus floor augmentation and the use of a grafting material.

Prosthetic rehabilitation in the atrophic posterior maxilla with a short implant has been proposed to preclude alveolar ridge augmentation (Figure 4). The definition of a short implant is inconsistent, and implant lengths between 6 mm and 10 mm have been defined as a short implant. Placement of short implants is associated with a reduced bone-to-implant contact and increased crown-to-implant ratio compared with placement of standard-length implants, which could lead to an increased risk of implant failure or loss. Systematic reviews comparing implant treatment outcomes following prosthetic rehabilitation of the atrophic posterior maxilla with short implant compared with sinus floor augmentation and standard-length implants have demonstrated comparable implant survival rates, risk of complications, and patient satisfaction [64,65,66,67,68,69,70,71]. However, the conclusions of the systematic reviews should be interpreted with caution due to study heterogeneity involving short implants with different lengths, comparison of splinted and non-splinted prosthetic solutions, and dissimilar observation periods.

A comprehensive review of the literature identified three randomized controlled trials assessing implant treatment outcomes following single-crown restorations supported by short implants (6 mm) compared with standard length implants (≥11 mm) in conjunction with sinus floor augmentation [72,73,74,75]. The observation period was one year [72,75] and five years, respectively [73,74]. One hundred and seventy-nine patients were included, and the total number of inserted implants was 228, of which 108 were short implants and 110 were standard-length implants. Survival of superstructures was 100% with both treatment methods after one year [72,73,74,75]. The implant survival was 95% and 98.5% for short implants compared with 100% for standard-length implants after five years [73,74]. The peri-implant marginal bone loss varied between 0.12 mm and 0.54 for short implants after five years [73,74]. Corresponding measurements for standard-length implants were 0.14 mm and 0.46 mm [73,74]. There were no significant differences in implant survival, marginal bone loss, and biological and technical complications between the two treatment modalities after five years [73,74]. Moreover, a significant improvement in oral-health-related quality of life and high patient satisfaction was reported with both treatment modalities after five years [73,74]. The association between crown-to-implant ratio and increased risk of implant failure or loss could not be proved [72,73]. It can, therefore, be concluded that a single-crown restoration supported by short implants seems to be an equivalent treatment method compared with the placement of standard-length implants in conjunction with sinus floor augmentation for prosthetic rehabilitation of the partially edentulous atrophic posterior maxilla. However, further long-term randomized controlled trials involving larger patient samples are required before definitive clinical implications can be assessed.

## 5. Vertical Ridge Augmentation—Sandwich versus Onlay Grafting

In the past, it has often been questioned whether a substantial vertical ridge augmentation with long-term stability is possible because a continuing jaw atrophy, especially in the absence of dental implants, was observed [4]. This section clarifies that a vertical ridge augmentation is possible and that the augmented ridge is stable. Furthermore, there is evidence that interpositional grafting is superior to onlay grafting for vertical augmentation, and whenever possible, this should be the method of choice.

**Figure 5:**
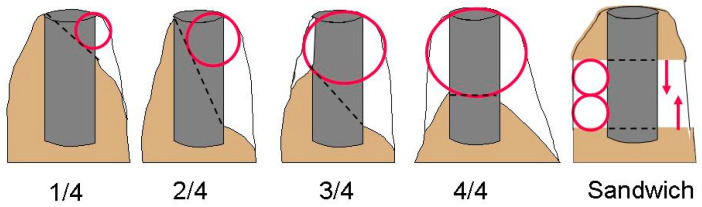
The quarter classification (Terheyden [76]) describes the bone deficit in an implant site in relation to the planned dental implant, starting with buccal bone loss of < 50% of the implant length (1/4), then buccal bone loss > 50% (2/4), then lingual/palatal bone loss < 50% (3/4) and finally loss of the alveolar crest buccal and palatal > 50% (4/4). The red circles indicate the required distance, which neoangiogenesis has to bridge to integrate a bone graft. The clinical limit is 3.7 mm. On the right side, it is shown that an interpositional graft between two vascularized bone surfaces doubles the bridging distance (7.4 mm), because blood vessels can grow from two directions as indicated by the red arrows.

To be functional, a non-vascularized bone graft must first become integrated and then remodeled by the process of neoangiogenesis within the bone graft. The new blood vessels grow into the graft from the base of the bone defect. Traditionally, onlay bone grafts were mainly used prior to implant placement. According to the quarter classification of bone deficits in the site of a dental implant (Terheyden [76]), the required amount of blood vessel growth increases with the defect class (Figure 5). New blood vessels grow into pores within the graft materials or take advantage of the spaces between particles of a particulate graft material. An interconnective porosity is required so that the arterial blood vessels can enter and the veins can return. It has been shown, on average, that with a particulate graft, 3.7 mm of newly vascularized bone can be achieved within 3–6 months [38]. The particulate graft beyond 3.7 mm is usually rejected or forms a hypovascular fibrous scar. Thus, neoangiogenesis is the limiting factor for how much bone graft height can be achieved. In interpositional situations, the bone graft is placed between two blood-perfused vital bone surfaces. Angiogenesis takes place from two sides, which firstly doubles the biological distance perfused and secondly decreases the time for graft incorporation. In clinical studies, approximately 7 mm vertical bone gain could be obtained instead of 3.7 mm (see further below). Preparing a sandwich interpositional graft bed involves creating a pedicled bone segment, which stays attached to the lingual or palatal soft tissues, remains perfused, and can be transported crestally and secured to create space for an interpositional graft.

Neoangiogenesis inside autogenous block grafts is enhanced by “creeping substitution” which involves “bone-cutting cones” similar to the remodeling of compact bone [76]. It has been reported that, on average, 5.8 mm of new bone can be substantially gained and remodeled within a few years [38]. For this reason, autologous block bone grafts became the traditional method for vertical augmentation of more than 3.7 mm. However, harvesting and transplantation of such a block graft is more invasive than the use of particulate autologous grafts or bone substitute materials. The sandwich interpositional technique offers the possibility of a significant vertical bone augmentation by placing a particulate graft, thus avoiding the harvesting of a block graft, which is more invasive and has the added advantage of being less of a surgical burden for the patient.

With onlay grafts, the need for primary coverage causes soft tissue tension when mobilizing the flap, which can result in wound dehiscence with partial or complete loss of the bone graft, which has been reported as high as 33–40% [77] On the other hand, the sandwich interpositional graft requires less flap mobilization. Although a 25% incidence of wound dehiscence is reported, the incidence of graft infection and rejection after sandwich grafting in the mandible was only 1.3% [78].

**Figure 6:**
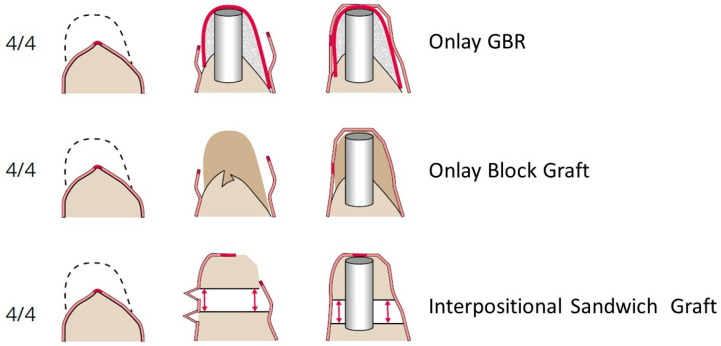
Different treatment options for a 4/4 defect. The dotted lines indicate the intended shape of the ridge after bone augmentation. The bold red part in the middle of the mucosal lining of the alveolar crest is the small area of fixed gingival tissue (masticatory gingiva). Due to the requirements of soft tissue coverage, the two onlay grafting options (GBR and block) cause a shift of the mucogingival junction, often requiring secondary soft tissue augmentation. In the interpositional grafting technique, the soft tissue on the lingual side stays attached to the bone segment, and this forms the new emergence profile of the dental implant. The implant is, therefore, covered from both sides with the original fixed gingival tissue. Very rarely, is secondary soft tissue augmentation needed.

Coverage of an onlay bone block or vertical guided bone regeneration (GBR) site requires the buccal tissue to be moved crestally, advanced over the graft in a palatal or lingual direction, and finally further stretched down apically to engage the pristine lingual/palatal mucosal margin. This triple flap mobilization usually ends with a palatal or lingual shift of the mucogingival border (Figure 6). The fixed gingiva moves beyond the emergence area of the subsequent dental implants, which accounts for the frequent need for secondary vestibuloplasties and soft tissue grafting [79]. However, in the case of sandwich grafting, the palatal or lingual soft tissues have never been detached from the bone segment and remain perfectly in place. Therefore, the emergence region of the dental implants is still covered with the original fixed mucosa. The superior soft tissue condition is a strong argument for sandwich interpositional bone augmentation.

Bone graft resorption is usually a surface resorption at denuded bone surfaces. During interpositional grafting, the lingual/palatal soft tissues stay attached to the transported bone segment [76]. Only the periosteum of the lingual or palatal flap has to be dissected to gain some freedom for the elevation of the transport segment. This is relatively easy in the mandible, but in the maxilla, the rigid palatal soft tissue allows only a vertical transport of 3–4 mm. The crestal soft tissues must not be detached from the transport segment because this would impede blood perfusion and result in surface resorption at the site where subsequent dental implants emerge. In sandwich grafting, the palatal or lingual soft tissues stay attached to the bone. Less graft resorption is, therefore, an argument in favor of sandwich interpositional grafting. If, finally, the bone graft has healed and has been taken by the organism, and if dental implants have successfully been placed, then the preserved fixed gingival tissue in the emergence area of the dental implants is an advantage for the sandwich method.

However, the limitations of interpositional grafting should be mentioned. When scar tissue or the rigid palatal mucoperiosteum allows only limited movements of the bone transport segment, distraction osteogenesis may be a better option [76]. The thin bone inferior to the maxillary sinus in the posterior maxilla is often a contraindication for segmental osteotomies, as well as a bone level of less than 4 mm superior to the alveolar nerve in the posterior mandible. In such situations, sinus grafting (inlay osteoplasty) or an onlay block graft in the mandible is indicated. However, in the posterior mandible with at least 4 mm or more residual bone superior to the nerve, the sandwich technique, even in a 3/4 defect with a knife edge ridge, is possible [76].

**Figure 7:**
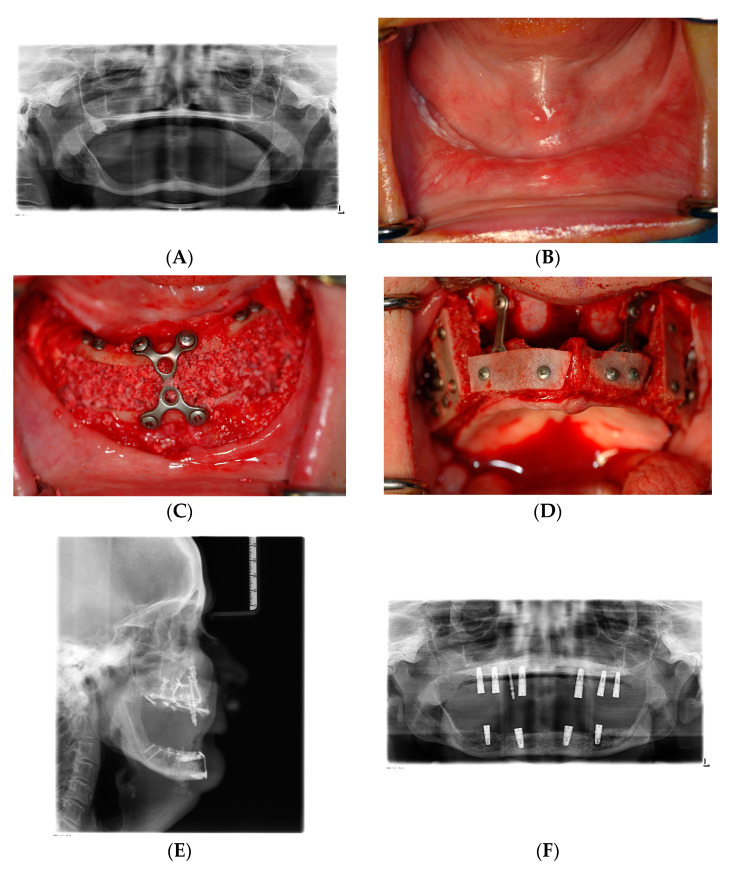

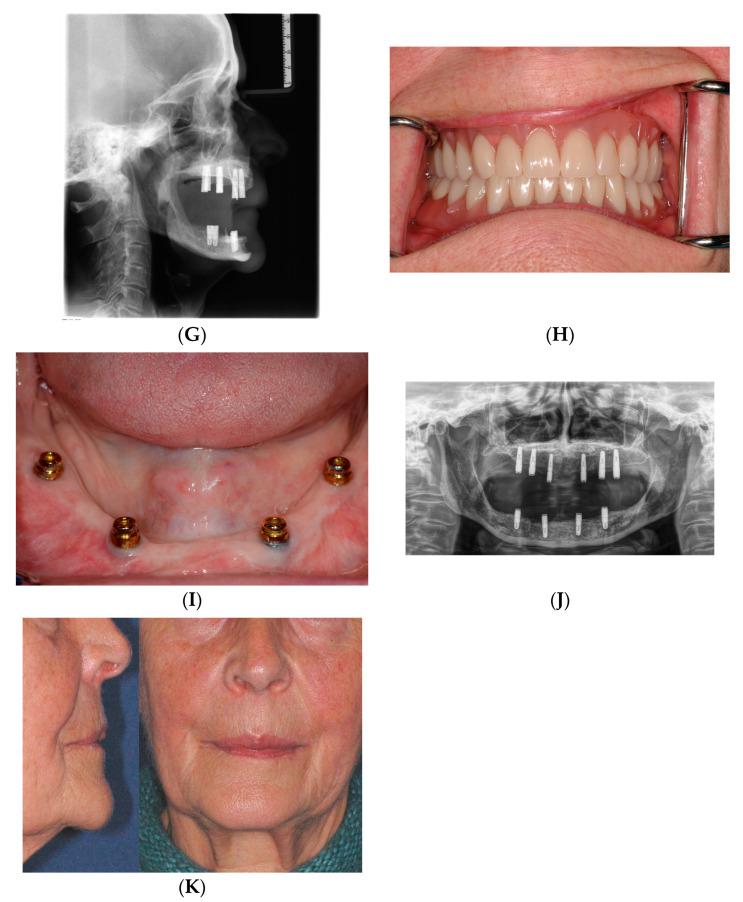
Vertical bone augmentation after severe alveolar atrophy in an edentulous 70-year-old female patient. (**A**) Severely atrophic maxilla and mandible (8 mm symphysis height). (**B**) Almost no fixed gingiva left; the floor of the mouth stands above the alveolar crest. (**C**) Intraoperative view. Sandwich osteotomy, space filled with mixed bone substitute. (**D**) Intraoperative view. Le Fort 1 interpositional grafting in the maxilla, supplemented with small strips of iliac block bone grafts. (**E**) Postoperative lateral cephalogram, vertical bone gain in maxilla and mandible. (**F**) Postoperative panoramic X-ray shows the utilization of the new bone for dental implants of regular length. (**G**) Lateral cephalogram, parallel positioning of the dental implants, no flaring of the implant axes in the maxilla. (**H**) Prosthetic treatment with full dentures on locator abutments. (**I**) Spontaneous re-appearance of the fixed gingival tissue without soft tissue augmentation of a vestibuloplasty (compare (**B**)). (**J**) Ten years later, almost no loss or resorption of the augmented bone in both jaws, the bone-protective effect of the dental implants. (**K**) Facial aesthetics: After vertical bone augmentation and prosthetic treatment, reduction of wrinkles and prominence of the vermilions, no drooling, adequate lower face height.

The principles of sandwich grafting of the atrophic mandible have been described in the era of classical preprosthetic surgery [80]. The edentulous atrophied mandible resorbed down to 8 mm symphysial height can be treated with a sandwich osteotomy and particulate graft [76] with almost zero bone loss over time after loading with endosteal dental implants (Figure 7A–J) [78]. Clinicians should not forget that such a bone augmentation in the extremely atrophic edentulous mandible enables not only placement of endosteal implants but also helps to prevent jaw fractures and restores the attachment for the facial muscles, improving the lip and chin position (Figure 7K). However, segmental osteotomies of the atrophic mandible carry the risk of a jaw fracture. For this reason, endosteal implants should be placed secondarily after 4 months.

In the mandible after sandwich osteoplasty, the reported height gain was 8.4 mm in the edentulous atrophic jaw and 4.8 mm in the partially dentate atrophic jaw posteriorly [78]. Implant survival was 96.7%, and success was 95.3% after 5 years. In this study, the long-term stability of the bone after placement and loading of dental implants was less than 0.1 mm, indicating long-term stability of the vertical augmentation over an observation time of 5–12 years [78]. In the atrophic edentulous maxilla, the Le Fort I interpositional grafting is equivalent to the sandwich osteoplasty of the edentulous mandible, and a similar stability of interpositional grafts has been observed in a study in the maxilla [81]. A vertical bone gain of 8.7 mm had been achieved, and over a 5-year observation period, long-term stability after placement of dental implants has been documented by 0.63 mm bone loss. In this retrospective study, implant survival after 5 years was 96.3%, and after 10 years, 95.9% following the Le Fort I osteotomy and interpositional grafting [81]. In the anterior maxilla, the sandwich method also has its place for vertical augmentation, even with simultaneous implant placement, and a bone gain of 4 mm has been reported in a randomized controlled trial [82]. Thus, long-term results after interpositional bone augmentation in combination with placement of dental implants demonstrate that the grafted bone remained stable under function, and the resorption has been stopped.

## 6. Conclusions

Modern preprosthetic surgery aims to provide functional and facial aesthetic rehabilitation for patients with complex situations. Preprosthetic surgery should assist general dentists and maxillofacial prosthodontists in a team approach in providing a not-always optimal but always adapted and responsible treatment concept.

According to Section 2, soft tissue grafting is not a replacement for bone grafting but an adjunctive measure. Bone is a key success factor for dental implants, and bone grafting should not be replaced by soft tissue grafting if a dental implant is not completely covered by bone.

According to Section 3, bone grafting enables not only the placement of dental implants but also provides support for a natural aesthetic gingiva, for the lips, and for the face, especially the facial mimic muscles. In contrast to bone grafts, prosthetic replacement of missing tissues only has a passive supportive function for the tissues and allows no insertion of active facial muscles or vital gingiva. In this aspect, patients who are fit for surgery should be informed about the option of bone reconstruction, even if that involves referral to another doctor or another institution.

According to Section 4, short dental implants of 6–10 mm length provide sufficient support for splinted and single crown fixed partial dentures. Bone augmentation of the maxillary floor can, therefore, be avoided in situations that allow the placement of short implants.

According to Section 5, in atrophic edentulous jaws and in shorter gap and free-end situations, modern interpositional grafting techniques allow reliable vertical bone augmentation, which remained stable over the years. Interpositional grafting should be preferred to onlay techniques.

Considering these results, it seems the age-old problem of resorption of the edentulous alveolar bone that plagued our forefathers [4] has been overcome by the functional loading of bone with endosteal implants with or without additional bone grafting and heralds the new era of predictable preprosthetic surgery.
